# Posterior composites and new caries on adjacent surfaces - any association? Longitudinal study with a split-mouth design

**DOI:** 10.1186/s12903-016-0167-2

**Published:** 2016-02-01

**Authors:** Rasa Skudutyte-Rysstad, Anne Bjørg Tveit, Ivar Espelid, Simen E. Kopperud

**Affiliations:** Faculty of Dentistry, University of Oslo, Norway, P. O. Box 1109, Blindern, NO-0317 Oslo, Norway; Nordic Institute of Dental Materials (NIOM), Sognsveien 70A, NO-0855 Oslo, Norway

**Keywords:** Dental caries, Dental restoration, Incidence, Dental health services, Follow-up study

## Abstract

**Background:**

The aim of this longitudinal study was to compare caries incidence in sound approximal surfaces adjacent to newly placed composite restorations with the caries incidence in corresponding surfaces in contralateral teeth without any restorations in contact; and to assess risk factors for dentine caries development on adjacent and control surfaces.

**Methods:**

Data from a practice-based study, where 4030 posterior approximal restorations placed in permanent teeth by clinicians working in a Public Dental Health Service in Norway, were used. The study was approved by the Regional Committee for Medical Research Ethics. The present study is based on a subsample of patients with a sound surface adjacent to a newly placed composite posterior approximal restoration. All individuals who had intact corresponding contralateral pairs of teeth in the same jaw, were included.

At the end of the follow-up period, the study restorations and their adjacent surfaces were evaluated clinically and radiographically. Status of the contralateral tooth pair at baseline and end point was based on recordings from routine dental examinations, retrospectively extracted from the electronic dental records.

**Results:**

One hundred and ninety three patients (mean age 15.0 years, SD = 3.4) met the inclusion criteria. The surfaces were followed on average for 4.8 years. Follow-up observations revealed that 41 % of adjacent surfaces remained sound, compared with 67 % of the control surfaces (*p <* 0.001). Restorations were placed in 17 % of adjacent surfaces, compared with 3 % of the control surfaces (*p <* 0.001). In multivariate logistic regression analysis adjacent surfaces in maxillary teeth had increased risk for dentine caries development (OR 3.1, CI 1.3–7.3).

**Conclusions:**

Caries incidence in intact approximal surfaces adjacent to newly placed composite posterior approximal restorations was significantly higher compared with the contralateral control surface without a restoration in contact. Adjacent surfaces in maxillary teeth had increased risk for dentine caries development.

## Background

Although there has been a shift towards less invasive operative treatment of approximal caries [1], placement of direct posterior approximal restorations is still a common procedure in daily dental practice.

Several studies have investigated the effect of restorations on periodontal health, and report that approximal restorations are associated with increased gingival bleeding and periodontal attachment loss [2–4]. However, less is known on whether a new restoration entails increased risk of caries on the adjacent tooth surface.

It has been suggested that operative treatment of approximal lesions eliminates caries challenge, not only for the tooth that is treated, but also for the approximal surface of the adjacent tooth [5]. On the other hand, restoration placement has also frequently been associated with damage to adjacent surfaces [6–8]. It has been reported that iatrogenic damage during cavity preparation for amalgam occurs in up to 60 % of surfaces neighboring Class II restorations [8]. According to Qvist et al. [8], 69 % of adjacent surfaces in permanent teeth had such damage, and damaged surfaces were restored four times as often as undamaged teeth within five years. Surface roughness of restorations, poor marginal adaptation can further contribute to increased plaque retention [9] and thus, increased caries risk. Moreover, it has been shown that the type of material in approximal contact with an adjacent untreated surface affects its caries risk, as caries progression is slower on surfaces in contact with fluoride releasing materials compared with amalgam [8, 10].

In Norway, a general ban on the use of mercury in dental products was imposed in 2008 and the vast majority of posterior restorations placed in dental practice are now of composite. The Academy of Operative Dentistry European Section (AODES) considers adhesively bonded resin composites to be “the material of choice” for use in posterior restorations [11]. However, to our knowledge, there are few clinical studies on caries development in surfaces adjacent to composite restorations. Considering that approximal restorations comprise a substantial proportion of all restorative treatment performed in daily dental practice, it is important to investigate whether placement of composite restorations affect the neighboring surfaces.

The aim of the present study was to compare caries incidence in approximal surfaces adjacent to newly placed composite posterior approximal restorations with caries incidence on contralateral surface without adjacent restorations and to assess risk factors for dentine caries development on adjacent and contralateral control surfaces.

## Methods

The present study used data from a practice-based study on the longevity of posterior dental restorations in Norway, where 4030 posterior approximal restorations placed by clinicians working in the Public Dental Health Service (PDHS) in Norway from 2001 to 2004 were followed up for more than four years [12]. In total, 27 dentists, 3 male and 24 female with a mean age 46.5 (SD = 8.9) years participated. The restorative materials used were four standard hybrid composites (Filtek Z100, Filtek Z250, Tetric Ceram and Herculite XRV) [12]. The dentists were not aware of the purpose of the present study a priori and used their standard routines regarding operative technique and choice of restorative material when placing the fillings. The study was approved by the Regional Committee for Medical Research Ethics South East, Norway, ID:18709. Written consent from the patients was obtained according to their directions.

For the present longitudinal study with a split-mouth design, a subsample of sound surfaces adjacent to approximal posterior restorations was selected. The inclusion criteria were as follows: patient receiving posterior approximal composite restoration in a permanent tooth, no clinically or radiographically detectable caries on the surface adjacent to the restoration, and a minimum follow-up period of four years from receiving a filling (baseline). To achieve a split-mouth control site, only patients with an intact contralateral pair of teeth in the opposite quadrant in the same jaw without clinically or radiographically detectable caries were selected. For example, for patients with a mesial composite restoration on tooth 36 and sound surface distally on tooth 35, both the mesial surface of tooth 46 and the distal surface of tooth 45 had to be sound in order to meet the inclusion criteria.

Data collection for the study included variables related to patient’s age, gender, caries experience, oral hygiene and tooth-related variables (tooth type, jaw and mouth side). Caries status of the restored surface and its adjacent surface was assessed clinically and radiographically by the dentist using a 5-grade scale described previously [13]. Grades 1 to 2 represent enamel and grades 3 to 5 dentine caries. This scoring system is routinely used for caries registration in the PDHS in Norway. Patients’ caries experience was recorded at the baseline as a sum of decayed (grades 3–5), missing (due to caries) and filled teeth (D_3-5_MFT) based on data in the electronic dental records (EDR). Assessment of oral hygiene was based on dentist’s clinical judgment and recorded as good, medium and poor. For statistical analyses oral hygiene was dichotomized into good versus medium/poor. At the end of the follow-up period, the study restorations and their adjacent surfaces were evaluated clinically and radiographically by the participating dentist. Status of the homologous contralateral tooth at baseline and endpoint, based on recordings from routine dental examinations (clinical and radiographic assessment) were retrospectively extracted from the EDR. During the study period, all the patients received regular preventive and restorative care at PDHS clinics.

Due to the practice-based nature of the present study, no formal calibration of the participating dentists was carried out, but the clinical procedures and documentation routines were introduced and discussed thoroughly during a two-day meeting.

In total, 204 surfaces in 193 patients (54 % female) met the inclusion criteria (mean age 15.0 years, SD = 3.4). For patients with several pairs of surfaces that met the inclusion criteria (*n =* 9), only one surface pair was randomly selected by flipping a coin, resulting in 193 pairs of approximal surfaces, one per patient.

### Statistical analyses

Data were analysed using the SPSS statistical program package (IBM SPSS 20.0, SPSS Inc., Chicago, IL, USA). Descriptive statistics and frequency distributions were used for comparing caries incidence on the test and control sites. Differences in proportions of sound surfaces adjacent to the composite restoration and on the split-mouth control surfaces during the follow-up period were compared by McNemar test. To determine risk factors associated with dentine caries development, two separate multivariate logistic regression analyses were performed; one for adjacent surfaces and one for the contralateral control group. Dentine caries was defined as development of caries grade 3–5 or restored during the observation period. Variables significant at *p* ≤ 0.2 level in bivariate analyses were entered into multivariate analysis. Results were reported using odds ratio (OR) and 95 % confidence interval (CI). A significance level of 5 % was used.

## Results

The mean caries experience of the participants measured as D_3-5_MFT was 5.3 (SD = 3.2). All the study surfaces were followed on average for 4.8 years (minimum 4, maximum 7 years). Follow-up observations revealed that 41 % of surfaces adjacent to composite remained sound compared with 67 % of control surfaces, *p <* 0.05 (Table 1). Moreover, surfaces adjacent to the composite restorations were five times more often restored, compared with the control surfaces (*p <* 0.05).

**Table 1:** Caries status of the approximal surface adjacent to the composite restoration and split-mouth control surface at follow-up

Surface	Adjacent to composite *N* (%)	Control *N* (%)
Sound	79 (41)	129 (67)*
Caries grade 1	31 (16)	16 (8)
Caries grade 2	42 (22)	26 (14)
Caries grade 3	5 (3)	13 (7)
Caries grade 4	2 (1)	3 (1)
Caries grade 5	1 (0)	0
Restored	33 (17)	6 (3)*
Total	193 (100)	193 (100)

*p < 0.05, McNemar test

The results of multivariate regression analysis for dentine caries development on adjacent and control surfaces are presented in Tables 2 and 3. In the bivariate analyses higher risk for dentine caries on surfaces adjacent to newly placed composite posterior approximal restorations was associated with having medium/poor oral hygiene, higher DMFT score and maxillary teeth (Table 2). In the multivariate logistic analysis adjacent surfaces in maxillary teeth had 3.1 times higher odds for dentine caries than adjacent surfaces in mandible. For contralateral surface without adjacent restoration, lower patient’s age and higher DMFT score were significantly associated with increased dentine caries risk in bivariate analyses (Table 3). After adjustment for oral hygiene in multivariate analyses, both age and DMFT score remained significantly associated with increased dentine caries risk on contralateral control surfaces.

**Table 2:** Risk factors associated with development of dentine caries on approximal surfaces adjacent to newly placed composite posterior approximal restorations

		Bivariate			Multivariate		
	% (*N*)	OR	95 % CI	*P*-value	OR	95 % CI	*P*-value
Age							
Continuous variable	100 (193)	1.0	0.9–1.1	0.97			
Gender							
Male	46 (88)						
Female	54 (105)	1.8	0.9–3.8	0.10			
Oral hygiene^a^							
Good	49 (94)						
Medium/poor	51 (96)	**2.6**	**1.2–5.3**	**0.01**	2.0	0.9–4.3	0.09
Caries experience (DMFT)							
Continuous variable	100 (193)	**1.1**	**1.0–1.3**	**0.02**	1.1	1.0–1.2	0.14
Tooth type							
Canine/Premolar	68 (131)						
Molar	32 (62)	1.9	0.9–3.9	0.07	1.4	0.6–3.2	0.38
Jaw							
Mandible	40 (78)						
Maxilla	60 (115)	**3.0**	**1.3–6.6**	**0.01**	**3.1**	**1.3–7.3**	**0.01**
Mouth side							
Left	52 (100)						
Right	48 (93)	1.2	0.6–2.3	0.66			

Results significant at 5 % level marked in bold

^a^Reduced N because of missing data

**Table 3:** Risk factors associated with development of dentine caries on contralateral control surfaces without restoration in contact

		Bivariate			Multivariate		
	% (*N*)	OR	95 % CI	*P*-value	OR	95 % CI	*P*-value
Age							
Continuous variable	100 (193)	**0.7**	**0.6–0.9**	**0.01**	**0.5**	**0.4–0.7**	**0.00**
Gender							
Male	46 (88)						
Female	54 (105)	0.8	0.3–2.0	0.66			
Oral hygiene^a^							
Good	49 (94)						
Medium/poor	51 (96)	1.8	0.7–4.6	0.2	1.0	0.4–3.1	0.95
Caries experience (DMFT)							
Continuous variable	100 (193)	**1.2**	**1.0–1.3**	**0.05**	**1.4**	**1.2–1.7**	**0.00**
Tooth type							
Canine/Premolar	68 (131)						
Molar	32 (62)	1.2	0.5–3.1	0.65			
Jaw							
Mandible	40 (78)						
Maxilla	60 (115)	1.2	0.5–3.1	0.68			
Mouth side							
Left	52 (100)						
Right	48 (93)	0.6	0.3–1.5	0.28			

Results significant at 5 % level marked in bold

^a^Reduced N because of missing data

## Discussion

This present study suggests that placement of composite restoration in an approximal surface is associated with increased caries and subsequent restoration risk in the adjacent tooth. Moreover, risk factors for dentine caries development were different for adjacent and contralateral control surfaces.

Findings in the present study are in accordance with the findings from a study by Qvist et al. [8], where operative treatment of approximal carious lesions with amalgam enhanced the need for restorative therapy of the adjacent teeth.

To our knowledge, this is the first study documenting caries incidence next to recently placed composite restorations. Because of its practice-based setting, the findings probably reflect the real life situation and thus may have important clinical implications. In the bivariate analysis, development of dentine caries on adjacent surfaces was significantly associated with patients’ caries experience, oral hygiene and maxillary teeth. In multivariate analyses, maxillary teeth remained significantly associated with caries on adjacent surfaces. The findings have several explanations. As previously reported, iatrogenic damage is a frequent side-effect of operative treatment of approximal carious lesions [6–8]. Moreover, adjacent teeth with preparation damage are more often restored compared with undamaged teeth [8]. In the present study adjacent surfaces in maxillary teeth had 3.1 times higher odds for dentine caries than adjacent surfaces in mandible. This finding supports increased possibility of caries due to preparation damage. Increased frequency of iatrogenic damage in maxillary teeth has been previously reported by Medeiros and Seddon [7]. One of the possible explanations to this finding is that chances of iatrogenic damage are higher in the upper jaw because of better visual access to the lower arch.

The higher restoration frequency in adjacent surfaces may be explained by the fact that damaged surfaces are more prone to mechanical plaque retention and therefore increased caries risk. It has also been suggested that enamel damage on approximal surfaces can be misdiagnosed as approximal radiolucencies due to caries, and result in unnecessary restorative treatment of caries free surfaces [7]. However, considering the change towards less invasive criteria for operative treatment of approximal caries in Norway [1, 14], this explanation for the present study seems less likely.

Another possible explanation is that there may be differences in bacterial colonisation and biofilm formation on dental restoration surface compared with enamel. Resin composite has been shown to have higher bacterial adhesion than human enamel [15, 16]. The surface roughness of the restoration is an additional factor that can lead to increased dental plaque accumulation [9, 17] and subsequently contribute to a more cariogenic environment in the interproximal space. Difficulties associated with re-establishing good contour and a tight contact when placing composite resin composite in approximal cavities may also have contributed to increased plaque accumulation and increased caries risk at the restored sites.

The differences in biofilm activity between the restoration and control site at baseline could also influence caries development, however, this was not considered in the present study.

In the present study, the surfaces adjacent to composite were more than five times more likely to be restored compared with control surfaces. The frequency of receiving restoration in the adjacent surface similar to that previously reported by Qvist et al. [8], where damaged permanent teeth required restorations four times as often as undamaged teeth within five years. The findings of both studies indicate that there is a higher risk for restoration of the neighbor tooth once one proximal surface has been restored.

Due to the split-mouth design in this practice-based longitudinal study, much of the inter-subject variability is removed, and the power of the study compared with the whole-mouth design is increased [18]. Because the sites were subjected to almost identical local environments, many potential confounding factors, such as diet and saliva, etc. are controlled for by study design [19]. Longitudinal study design is considered an additional strength, since both caries and restoration incidence estimates could be calculated.

The present study has several limitations that should be considered when interpreting the results. Due to the practice-based nature of the present study, no formal calibration of the participating dentists was carried out. Reliability measures for diagnostic differences between participating clinicians were not available and magnitude of possible detection bias is therefore unknown. On the other hand, it has been shown that caries data collected from public health records are not decisively inferior compared to those obtained from examinations by trained and calibrated examiners [20]. One of the possible explanations might be that PDHS have detailed, nationally uniform instructions for diagnostic and treatment procedures as well as regular calibration meetings for their staff.

Caries status of the adjacent surfaces was assessed clinically and radiographically before the placement of composite restoration and radiographically at the end of the follow-up. Although the adjacent surface was registered as sound before restoration placement, one can speculate whether due to the possible differences in bacterial colonisation and biofilm formation during the period when the neighboring surface was developing caries, the enamel of adjacent surface had been exposed to a greater caries risk compared to the control surface [21]. Due to the practice-based nature of the study, site-specific dental plaque scorings were not performed, and therefore it was not possible to control for differences in oral hygiene between test and control sites. Another limitation is a possibility that some amount of enamel demineralisation on the adjacent surface could be present but not detected at baseline. This is because a certain amount of demineralisation has to be present before a lesion can be detected radiographically [22]. Moreover, radiographic diagnosis is unreliable for small carious lesions [23] and the radiographic image may detect varying signs of caries depending on the direction of the X-ray beam. The fact that the adjacent surface was available for direct visual-tactile assessment before the filling was placed would probably reduce the possibility of under-diagnosis of early enamel lesions on the adjacent surfaces to some extent.

Within the limitations of the present study, the results indicate that composite restoration placement in an approximal surface enhances the need for future operative treatment in many adjacent tooth surfaces. This might be considered as an important adverse effect of posterior approximal composite restorations which needs to be further investigated. The present study did not attempt to determine whether the increased caries incidence was attributable to the iatrogenic damage or restoration quality. More studies are warranted to assess factors of importance on caries development in teeth adjacent to composite restorations.

## Conclusions

Caries incidence in intact approximal surfaces adjacent to newly placed composite posterior approximal restorations was significantly higher compared with the contralateral control surface without a restoration in contact. Operative treatment of unrestored approximal surfaces should be as conservative as possible and cautious in respect to the neighboring surface. The findings indicate that interproximal surfaces in contact with a composite restored surface may require intensified preventive regimes as well as close monitoring and follow-up.
